# Association between sperm mitochondrial DNA copy number and deletion rate and industrial air pollution dynamics

**DOI:** 10.1038/s41598-022-12328-9

**Published:** 2022-05-18

**Authors:** Miluse Vozdova, Svatava Kubickova, Vera Kopecka, Jaroslav Sipek, Jiri Rubes

**Affiliations:** Department of Genetics and Reproductive Biotechnologies, Central European Institute of Technology - Veterinary Research Institute, Hudcova 70, 621 00 Brno, Czech Republic

**Keywords:** Genetics, Risk factors, Environmental impact

## Abstract

The effects of air pollution on men’s reproductive health can be monitored by evaluating semen quality and sperm DNA damage. We used real-time PCR to analyse the effects of air pollution on sperm mitochondrial DNA copy number (mtDNAcn) and deletion (mtDNAdel) rates in semen samples collected from 54 men in two seasons with different levels of industrial and traffic air pollution. MtDNAdel rates were significantly higher following the high exposure period and were positively correlated with mtDNAcn. However, we did not find any difference in mtDNAcn between the two seasons. MtDNAcn was positively correlated with the DNA fragmentation index and the rates of sperm with chromatin condensation defects, previously assessed by sperm chromatin structure assay, and negatively correlated with sperm concentration, progressive motility, viability, and normal morphology. This indicates that mtDNAcn is more closely associated with male fertility than mtDNAdel rates. In contrast, mtDNAdel might be a more sensitive biomarker of air pollution exposure in urban industrial environments.

## Introduction

Given growing concerns about human infertility, male reproductive health and sperm quality have received increasing attention in many developed countries. Continued industrialization and associated exposure to environmental pollutants pose serious risks for spermatogenesis and the production of normal sperm capable of fertilizing oocytes. High levels of air pollution have been associated with reduced sperm concentrations, motility and normal sperm morphology^1–3^, increased sperm chromatin fragmentation and changes in sperm DNA methylation^4–7^. In addition, environmental contaminants can also stand behind increased sperm mitochondrial DNA deletion (mtDNAdel) rates and changes in mtDNA copy number (mtDNAcn)^8,9^. A combination of standard and molecular semen analysis thus serves as a sensitive tool for assessing the impact of air pollution on human health^10,11^.

Mitochondrial status is closely related to sperm functionality. Mitochondria form a mitochondrial envelope located in the junction (midpiece) of the sperm tail^12–14^. Glycolysis and oxidative phosphorylation, which produce energy crucial for sperm cellular homeostasis and motility, are a key function of mitochondria^15,16^. The mitochondrial genome is 16.6 kb long, circular, and encodes several genes primarily involved in energy metabolism and protein synthesis^17^. Due to the absence of protective histones and a lack of efficient DNA repair mechanisms, mtDNA is susceptible to damage caused by reactive oxygen species (ROS) generated during mitochondrial oxidative phosphorylation or associated with environmental exposure^13,18,19^. Analysis of mtDNA copy number and rates of mutation and deletion are useful not only for male fertility assessments but also in monitoring oxidative stress and environmental exposure^8,9,20–25^.

In the Czech Republic, the city of Ostrava and its surrounding area are severely affected by air pollution produced by the heavy iron industry, coke oven plants, local combustion and traffic. The region has some of the highest concentrations of particulate matter (PM_10_ and PM_2.5_), benzene and, in particular, benzo[a]pyrene (B[a]P) in Europe^21^. The air in this region exhibits periods of high pollution in winter, followed by significantly lower levels of pollution in summer^27,28^. Studies have shown that air pollution causes increased postneonatal infant mortality and bronchitis in children and adults in Ostrava^21,29^.

The aim of this preliminary study was to analyse the possible effects of seasonal changes in air pollution on sperm mtDNAcn and mtDNAdel rates in men living and working in the industrial urban agglomeration. The study is a part of the “Healthy Ageing in the Industrial Environment” (HAIE) project, which focuses on the health and reproductive consequences of air pollution. MtDNA of semen samples collected from men following "high" (winter season) and "low" (summer season) exposure were analysed in association with previously obtained data on semen quality and sperm chromatin integrity^5^.

## Material and methods

### Samples

Semen samples were obtained from 54 healthy nonsmoking municipal policemen living and working in Ostrava (Czech Republic) who were exposed to air pollutants on a daily basis while patrolling in the city streets on their regular shifts. They spent 80% of their daily working time in both winter and summer patrolling on foot. The study group consisted of nonsmokers 40.4 ± 9.4 years old (range: 21–61 years). Their reproductive and general health and factors that might have affected their semen quality were assessed with a questionnaire. The obtained data did not show any exceptional conditions or health and reproductive issues. The subjects reported only moderate alcohol consumption, no drug abuse and no home exposure to chemical toxicants. All of them reported a mixed diet, including the consumption of meat, in both seasons. The study was conducted according to the guidelines of the Declaration of Helsinki and approved by the Ethics Committee of the Institute of Experimental Medicine, Academy of Sciences of the Czech Republic (ASCR) in Prague (approval number: 2018/09). Written informed consent was obtained from all subjects involved in the study.

As spermatogenesis lasts 72–90 days, semen samples were collected in March and October 2019 following the period of high (winter) and low (summer) air pollution exposure, respectively. The concentrations of the main air pollutants in the two seasons were detected by fixed air pollution sensors run by the Czech Hydrometeorological Institute. Concentrations of PM_2.5_, PM_10_, and NO_2_ were measured hourly by automatic emission monitoring, and the results of the one-hour measurements were processed to obtain daily averages. Benzene was measured as a 24-h sample every two weeks. A 24-h sample was evaluated for the B[a]P measurements every third day.

### Semen analysis

Semen samples were collected by masturbation after 2–7 days of sexual abstinence and allowed to liquefy at room temperature. Standard semen parameters (semen volume, sperm concentration, motility, morphology, and viability) were assessed in accordance with the World Health Organization (WHO) guidelines^30^. Briefly, sperm counts were determined using a Bürker chamber, sperm motility was evaluated under a light microscope at 200 × magnification, and sperm viability was assessed as the percentage of sperm without plasma membrane damage detected by staining with eosin-nigrosin. The percentage of morphologically normal sperm was determined by an evaluation of 200 sperm at 1000 × magnification after staining with a Diff-Quik rapid staining kit. Strict scoring criteria described by the WHO (2010) were applied. Sperm DNA damage was analysed by the Sperm Chromatin Structure Assay (SCSA) assessing the rates of sperm with fragmented DNA (DNA fragmentation index, DFI) and sperm with high-density staining (HDS, immature sperm) as previously described^31^. Then, the semen samples were aliquoted, cooled and transported to our laboratory.

### Sample processing and DNA isolation

The semen aliquots intended for mtDNA analysis were processed using GuEX buffer within 24 h. Briefly, the samples were washed in PBS, and the sediment was processed using 400 µl of GuEX buffer (50 mM guanidine hydrochloride, 10.5 mM Tris pH 8.0, 10.5 mM NaCl, 10.5 mM EDTA pH 8.0, 1 mM NaOH, pH 8.0–8.5; Sigma, St. Louis, MO, USA) and 20 µl of proteinase K (20 mg/ml) (Qiagen, Hilden, Germany) for 15 min at 37 °C. After centrifugation at 6600 rpm for 10 min, the sediment was resuspended in 200 µl of PBS. The processed sperm samples were frozen until analysis. Sperm genomic DNA was isolated using the QIAamp DNA Blood Mini Kit (Qiagen, Hilden, Germany) with 30 µl of 1 M dithiothreitol (Sigma) and stored frozen.

### MtDNA copy number and deletion analysis by real-time PCR

MtDNAcn and mtDNAdel rates were assessed using real-time PCR^18^. A single copy nuclear locus (beta-2 microglobulin, *β2M*), the invariable mtDNA MinorArc, and the mtDNA MajorArc comprising most of the known deletion sites (https://www.mitomap.org) were targeted in three separate reactions using primers displayed in Table 1.

**Table 1:** PCR primers used in this study.

Target	Primer sequence (5′–3′)	Position	Product length
mtMinArc	CTAAATAGCCCACACGTTCCC	mt:16,528–16,548	84 bp
	AGAGCTCCCGTGAGTGGTTA	mt:23–42	
mtMajArc	CAACCTTTTCCTCCGACCC	mt:10,920–10,938	98 bp
	ACTGGATAAGTGGCGTTGGC	mt:10,998–11,017	
*β2M*	GCTGGGTAGCTCTAAACAATGTATTCA	Chr15:15,798,932–15,798,958	94 bp
	CCATGTACTAACAAATGTCTAAAATGGT	Chr15:15,798,999–15,799,026	

All real-time PCR assays were performed in triplicate with 10 µl containing 1 × SYTO-9 Master Mix (Top–Bio, Prague, Czech Republic), 0.3 μM primers and 10 ng of genomic DNA. Real-time PCR was performed using the following conditions: 95 °C for 4 min and 40 cycles of 94 °C for 60 s, 55 °C for 30 s and 72 °C for 45 s on the CFX96 Touch Real-time PCR Detection System (BioRad, Hercules, CA, USA). The melting curves were assessed at 55–95 °C. The amplification efficiencies evaluated using six-point standard curves were 95% for *β2M* and MinorArc and 93% for MajorArc.

### Statistical analysis

Statistical analysis of the results was performed by nonparametric exact tests using SPSS software, version 18 for Windows (SPSS, Inc. Chicago, IL, USA). Paired Wilcoxon signed ranks test was used to compare mtDNAcn and mtDNAdel rates between the two sampling periods. Partial Spearman’s correlations were used to analyse the relationships among mtDNAcn, mtDNAdel rates, semen quality and chromatin integrity, adjusted to age.

## Results

In 2019, the first and third quarterly average concentrations of the pollutants in the whole territory of Ostrava were 32.8 ± 2.8 µg/m^3^ and 19.5 ± 1.6 µg/m^3^ PM_10_, 27.7 ± 2.3 µg/m^3^ and 14.5 ± 1.2 µg/m^3^ PM_2.5_, 19.7 ± 4.0 µg/m^3^ and 12.5 ± 2.6 µg/m^3^ NO_2_, 2.4 ± 0.4 µg/m^3^ and 1.3 ± 0.3 µg/m^3^ benzene, and 4.6 ± 1.3 ng/m^3^ and 0.6 ± 0.3 ng/m^3^ B[a]P, respectively. The concentrations of all monitored pollutants were significantly higher during the winter season (P < 0.001).

The results of the semen and chromatin integrity (sperm chromatin structure assay; SCSA) analyses are shown in Supplementary Table S1. There were no significant differences in semen quality between the spring and autumn sampling periods, except sperm motility, which was significantly lower in autumn. The DFI and rates of HDS sperm were significantly higher in spring, following the high air pollution period^5^.

**Figure 1:**
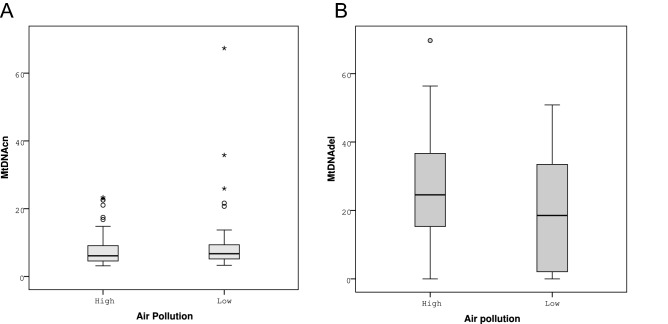
Comparison of sperm mtDNAcn (**A**) and mtDNAdel rates (**B**) following the high (winter) and low (summer) air pollution periods. The vertical height of each box represents the range of 25–75% of the data (the interquartile range; IQR), the horizontal line within each box represents the median value, and the upper and lower extensions represent the largest and smallest values that were determined to not be outliers. The open circles (○) denote simple outliers, i.e., samples that fell more than > 1.5 IQR from the 25th percentile of the distribution, whereas asterisks (*) denote extreme outliers, i.e., samples that fell > 3 IQR from the 25th percentile of the distribution.

The results of the mtDNA analysis are displayed in Fig. 1 and Supplementary Table S1. We did not detect any significant difference in mtDNAcn between the spring and autumn semen collection (P = 0.247). MtDNAdel rates were significantly higher in spring, following the high exposure period (P = 0.049).

As displayed in Table 2, mtDNAcn was significantly negatively correlated with sperm concentration, progressive motility, and normal morphology rates in both collection seasons and was significantly negatively correlated with sperm viability in spring. Additionally, mtDNAcn was significantly positively correlated with the mtDNAdel rate in spring and with the DFI and HDS rates in autumn. We did not detect any correlation between mtDNAdel rates and semen parameters or chromatin integrity. Neither mtDNAcn nor mtDNAdel rates showed any significant association with age.

**Table 2:** Spearman’s correlation of mtDNAcn and mtDNAdel rate with semen parameters and sperm chromatin characteristics following periods of high and low air pollution, adjusted for age.

	MtDNAcn				MtDNAdel			
	High pollution		Low pollution		High pollution		Low pollution	
	Rho	P	Rho	P	Rho	P	Rho	P
Concentration	−0.321*	0.019	−0.364**	0.007	0.227	0.103	0.215	0.123
Progressive motility	−0.280*	0.043	−0.278*	0.044	0.110	0.434	0.058	0.679
Morphology	−0.347*	0.011	−0.296*	0.031	−0.149	0.288	−0.228	0.100
Viability	−0.473**	< 0.001	−0.251	0.070	−0.053	0.705	−0.083	0.557
SCSA-DFI	0.269	0.051	0.461**	< 0.001	−0.104	0.461	0.252	0.068
SCSA-HDS	0.270	0.051	0.419**	0.002	−0.199	0.154	−0.029	0.839
MtDNAdel	0.425**	0.001	0.158	0.258	1	–	1	–
MtDNAcn	1	–	1	–	0.425**	0.001	0.158	0.258

**Correlation is significant at the 0.01 level. *Correlation is significant at the 0.05 level.

## Discussion

Air pollution in the Ostrava industrial region consists of particulate matter, as well as gaseous pollutants. In particular, the concentrations of polycyclic aromatic hydrocarbons (PAHs) regularly exceed acceptable thresholds^21^. In 2019, the Czech Hydrometeorological Institute in Ostrava reported quarterly average concentration of B[a]P emissions in winter (4.6 ng/m^3^) that were four times higher than the yearly national limit of 1.0 ng/m^3^ (Czech law No. 369/2016).

Exposure to industrial air pollution is a known risk factor for male reproduction. It can affect sperm concentration, motility, morphology, sperm chromatin integrity and DNA methylation^1–7^. Additionally, sperm mtDNA is susceptible to induced changes; sperm mtDNAcn and mtDNAdel rates are emerging biomarkers of environmental exposure^8,9,32^.

Although the responsiveness of mtDNA biomarkers to environmental exposure has previously been reported, the data are still contradictory. Higher mtDNAcn was previously detected in the peripheral blood of workers chronically exposed to PAHs^33^. In contrast, other authors observed decreased mtDNAcn in peripheral blood in association with exposure to PAHs^34,35^. Increased blood mtDNAcn was also found associated with traffic-related air pollution^36,37^ and exposure to benzene^38^. However, decreased blood mtDNAcn was detected in individuals exposed to traffic-generated particulate matter in another study^39^. Moreover, regarding sperm mtDNA, decreased sperm mtDNAcn was reported after exposure to PAHs^32^, but other studies found a positive association of sperm mtDNAcn with air pollution and urinary monocarboxy-isononyl phthalate concentrations^8,9^. No significant relationships between air pollution exposure and sperm mtDNAcn and mtDNA integrity were reported by other authors^40^. Similarly, no correlation was detected between sperm MtDNA integrity and exposure to PAHs or phtalates^8,32^. In this context, also the dynamics and time course of mtDNA changes require further exploration. However, such studies, including evaluations of mtDNA characteristics in repeated samples from the same subjects, are lacking.

In the current preliminary study, we analysed successive semen samples collected from a group of healthy men to identify potential differences in sperm mtDNAcn and mtDNAdel rates between two seasons that had different levels of industrial and traffic air pollution. Our method of repeated sampling of the same men (instead of comparing different study groups) allowed us to minimize any effects of internal and lifestyle factors, including smoking, diet and consumption of supplements, as well as specific gene interactions on sperm mtDNA. Additionally, we enrolled only nonsmokers in this study, and the study group was homogeneous regarding profession and time spent outdoors, moderate alcohol consumption, and absence of drug abuse or additional exposure to chemical toxicants. According to the questionnaires, the subjects did not exhibit any change in diet, lifestyle or exposure in the period covered in this study.

The sperm mtDNAcn values did not significantly differ between the two seasons. However, we detected significantly higher mtDNAdel rates following high air pollution exposure during winter. MtDNAcn was positively correlated with mtDNAdel rates in spring, negatively correlated with most semen parameters, and positively correlated with the sperm chromatin fragmentation (DFI) and HDS rates. These findings are in agreement with those of other papers reporting significantly elevated mtDNAcn in infertile men with abnormal semen parameters^21,41^. Higher sperm mtDNAcn is associated with a lower probability of achieving pregnancy within 12 months and a longer time to pregnancy^24^. Additionally, increased mtDNAdel rates were previously reported to be associated with a decline in sperm motility and fertility^20,21^. Nevertheless, such a correlation was not found in our preliminary study.

It is not surprising that the quality of sperm mitochondria and mtDNA are closely related to fertility. Mitochondria produce the energy required for sperm motility, which is one of the main features that characterize fertile sperm. However, mitochondrial biochemical activity can result in extensive ROS formation associated with increased oxidative stress and its consequences. ROS generated by mitochondrial lipid peroxidation play an important role in sperm physiology and function by inducing sperm hyperactivation, capacitation, the acrosome reaction and binding to the zona pellucida^42^. Nevertheless, increased ROS formation and oxidative stress negatively influence sperm chromatin condensation during maturation, impair sperm motility and viability, and induce DNA damage^42–47^. This and the previously reported association between mtDNAcn and ROS levels^45^ can explain the correlations between mtDNAcn and semen parameters, abnormal chromatin condensation (HDS rate), nuclear DNA fragmentation (DFI), and mtDNAdel rates in our study. Considering the relative stability of mtDNAcn in the two analysed seasons, the observed increase in mtDNAdel rates in the spring can be attributed to the seasonal increase in oxidative stress resulting from high air pollution exposure in winter. However, we cannot exclude the possible roles of other factors. For example, the effects of ambient and scrotal temperature, daylight length and associated hormonal levels must be further investigated. Such factors probably play a role in the previously described natural seasonal changes in semen quality, which can be characterized by improved semen parameters in winter and spring followed by lower sperm concentrations and progressive motility rates in summer and autumn, the latter of which was observed also in this study^48–51^.

## Conclusions

In this preliminary study, we showed an association between high sperm mtDNAcn and lower semen parameters, and with the sperm chromatin and mtDNA damage. Nevertheless, mtDNAcn remained constant between the two sampling periods; thus, mtDNAdel rate appears to be a more sensitive biomarker of seasonal changes in air pollution exposure in urban industrial environments.

## Supplementary Information

Supplementary Information.

## Data Availability

Data on mtDNA is contained within this article. Data on the sperm quality, DFI and HDS rates used for the correlation analysis in this study were previously published^5^, and their summary is available in Supplementary Table S1.

## References

[1] Selevan SG (2000). Semen quality and reproductive health of young Czech men exposed to seasonal air pollution. Environ. Health Perspect..

[2] Hammoud A (2010). Decreased sperm motility is associated with air pollution in Salt Lake City. Fertil. Steril..

[3] Santi D (2018). Seasonal variation of semen parameters correlates with environmental temperature and air pollution: A big data analysis over 6 years. Environ. Pollut..

[4] Rubes J (2005). Episodic air pollution is associated with increased DNA fragmentation in human sperm without other changes in semen quality. Hum. Reprod..

[5] Rubes J, Sipek J, Kopecka V, Musilova P, Vozdova M (2021). Semen quality and sperm DNA integrity in city policemen exposed to polluted air in an urban industrial agglomeration. Int. J. Hyg. Environ. Health.

[6] Consales C (2016). Exposure to persistent organic pollutants and sperm DNA methylation changes in Arctic and European populations. Environ. Mol. Mutagen..

[7] Ma Y, Lu Z, Wang L, Qiang M (2019). Correlation of internal exposure levels of polycyclic aromatic hydrocarbons to methylation of imprinting genes of sperm DNA. Int. J. Environ. Res. Public Health.

[8] Huffman AM (2018). Associations of urinary phthalate metabolites and lipid peroxidation with sperm mitochondrial DNA copy number and deletions. Environ. Res..

[9] Zhou L (2021). Sperm mtDNA copy number, telomere length, and seminal spermatogenic cells in relation to ambient air pollution: Results of a cross-sectional study in Jing-Jin-Ji region of China. J. Hazard. Mater..

[10] Montano, L., Bergamo, P. & Lorenzetti, M. G. A. and S. The Role of Human Semen as an Early and Reliable Tool of Environmental Impact Assessment on Human Health. In *Spermatozoa - Facts and Perspectives*. IntechOpen. 10.5772/intechopen.73231 (2018).

[11] Bergamo P (2016). Human semen as an early, sensitive biomarker of highly polluted living environment in healthy men: A pilot biomonitoring study on trace elements in blood and semen and their relationship with sperm quality and RedOx status. Reprod. Toxicol..

[12] Sutovsky P, Navara CS, Schatten G (1996). Fate of the sperm mitochondria, and the incorporation, conversion, and disassembly of the sperm tail structures during bovine fertilization. Biol. Reprod..

[13] Rajender S, Rahul P, Mahdi AA (2010). Mitochondria, spermatogenesis and male infertility. Mitochondrion.

[14] Lindemann CB, Lesich KA (2016). Functional anatomy of the mammalian sperm flagellum. Cytoskeleton.

[15] Nascimento JM (2008). Comparison of glycolysis and oxidative phosphorylation as energy sources for mammalian sperm motility, using the combination of fluorescence imaging, laser tweezers, and real-time automated tracking and trapping. J. Cell. Physiol..

[16] Losano JDA (2018). Spermatic mitochondria: Role in oxidative homeostasis, sperm function and possible tools for their assessment. Zygote.

[17] Anderson S (1981). Sequence and organization of the human mitochondrial genome. Nature.

[18] Phillips NR, Sprouse ML, Roby RK (2014). Simultaneous quantification of mitochondrial DNA copy number and deletion ratio: A multiplex real-time PCR assay. Sci. Rep..

[19] Leni Z, Künzi L, Geiser M (2020). Air pollution causing oxidative stress. Curr. Opin. Toxicol..

[20] Kao SH, Chao HT, Wei YH (1998). Multiple deletions of mitochondrial DNA are associated with the decline of motility and fertility of human spermatozoa. Mol. Hum. Reprod..

[21] Karimian M, Babae IF (2020). Large-scale mtDNA deletions as genetic biomarkers for susceptibility to male infertility: A systematic review and meta-analysis. Int. J. Biol. Macromol..

[22] Song GJ, Lewis V (2008). Mitochondrial DNA integrity and copy number in sperm from infertile men. Fertil. Steril..

[23] Sutovsky P, Lovercamp K (2010). Molecular markers of sperm quality. Soc. Reprod. Fertil. Suppl..

[24] Wu H (2019). Associations of sperm mitochondrial DNA copy number and deletion rate with fertilization and embryo development in a clinical setting. Hum. Reprod..

[25] Rosati AJ (2020). Sperm mitochondrial DNA biomarkers and couple fecundity. Hum. Reprod..

[26] Jirik V (2016). Air pollution and potential health risk in Ostrava Region—A review. Cent. Eur. J. Public Health.

[27] Cernikovsky L, Krejci B, Blazek Z, Volna V (2016). Transboundary air-pollution transport in the Czech-Polish border region between the cities of Ostrava and Katowice. Cent. Eur. J. Public Health.

[28] Svedova B (2020). Concentration variability of water-soluble ions during the acceptable and exceeded pollution in an industrial region. Int. J. Environ. Res. Public Health.

[29] Tomaskova H (2016). PM10 air pollution and acute hospital admissions for cardiovascular and respiratory causes in Ostrava. Cent. Eur. J. Public Health.

[30] World Health Organization (2010). WHO Laboratory Manual for the Examination and Processing of Human Semen.

[31] Rubes J (2010). Genetic polymorphisms influence the susceptibility of men to sperm DNA damage associated with exposure to air pollution. Mutat. Res..

[32] Ling X (2017). Polycyclic aromatic hydrocarbons exposure decreased sperm mitochondrial DNA copy number: A cross-sectional study (MARHCS) in Chongqing, China. Environ. Pollut..

[33] Pavanello S (2013). Mitochondrial DNA copy number and exposure to polycyclic aromatic hydrocarbons. Cancer Epidemiol. Biomark. Prev..

[34] Pieters N (2013). Decreased mitochondrial DNA content in association with exposure to polycyclic aromatic hydrocarbons in house dust during wintertime: From a population enquiry to cell culture. PLoS ONE.

[35] Zhao X (2020). Reduction of mitochondrial DNA copy number in peripheral blood is related to polycyclic aromatic hydrocarbons exposure in coke oven workers: Bayesian kernel machine regression. Environ. Pollut..

[36] Hou L (2010). Airborne particulate matter and mitochondrial damage: A cross-sectional study. Environ. Health.

[37] Zhong J (2016). Traffic-related air pollution, blood pressure, and adaptive response of mitochondrial abundance. Circulation.

[38] Carugno M (2012). Increased mitochondrial DNA copy number in occupations associated with low-dose benzene exposure. Environ. Health Perspect..

[39] Hou L (2013). Inhalable particulate matter and mitochondrial DNA copy number in highly exposed individuals in Beijing, China: A repeated-measure study. Part. Fibre Toxicol..

[40] Zhang G (2020). Associations of ambient air pollutant exposure with seminal plasma MDA, sperm mtDNA copy number, and mtDNA integrity. Environ. Int..

[41] May-Panloup P (2003). Increased sperm mitochondrial DNA content in male infertility. Hum. Reprod..

[42] de Lamirande E, Jiang H, Zini A, Kodama H, Gagnon C (1997). Reactive oxygen species and sperm physiology. Rev. Reprod..

[43] Cocuzza M (2008). Age-related increase of reactive oxygen species in neat semen in healthy fertile men. Urology.

[44] Abasalt HC, Gholamali JS, Maryam GC (2013). Lipid peroxidation and large-scale deletions of mitochondrial DNA in asthenoteratozoospermic patients. Indian J. Biochem. Biophys..

[45] Bonanno O (2016). Sperm of patients with severe asthenozoospermia show biochemical, molecular and genomic alterations. Reproduction.

[46] Dorostghoal M, Kazeminejad SR, Shahbazian N, Pourmehdi M, Jabbari A (2017). Oxidative stress status and sperm DNA fragmentation in fertile and infertile men. Andrologia.

[47] Berby B (2021). Oxidative stress is associated with telomere interaction impairment and chromatin condensation defects in spermatozoa of infertile males. Antioxidants.

[48] Levitas E, Lunenfeld E, Weisz N, Friger M, Har-Vardi I (2013). Seasonal variations of human sperm cells among 6455 semen samples: A plausible explanation of a seasonal birth pattern. Am. J. Obstet. Gynecol..

[49] Kabukçu C (2020). Do seasonal variations in ambient temperature, humidity and daylight duration affect semen parameters? A retrospective analysis over eight years. Andrologia.

[50] Mao H, Feng L, Yang W-X (2017). Environmental factors contributed to circannual rhythm of semen quality. Chronobiol. Int..

[51] Wang X (2020). The association between ambient temperature and sperm quality in Wuhan, China. Environ. Health.

